# Translation and validation of the greek version of a questionnaire measuring patient views on participation in clinical trials

**DOI:** 10.1186/s12913-021-07111-x

**Published:** 2021-10-22

**Authors:** Dimitrios Karampatakis, Angeliki Kakavouti-Doudos, Panagiotis Oikonomidis, Polychronis Voultsos

**Affiliations:** 1 1st University Eye Clinic, School of Medicine, Faculty of Health Sciences, Aristotle University, University Campus, 541 24 Thessaloniki, Greece; 2 Laboratory of Forensic Medicine & Toxicology (Medical Law and Ethics), School of Medicine, Faculty of Health Sciences, Aristotle University, University Campus, 541 24 Thessaloniki, Greece

**Keywords:** Clinical trial participation, Questionnaire / scale, Barriers, Facilitators, Greece / Greek

## Abstract

**Background:**

The increasing number of clinical research opportunities requires increasing numbers of participants in clinical trials. However, it may become increasingly problematic, as protocols have become increasingly complex. Better understanding of patients’ attitudes towards their potential participation in clinical trials is essential for developing effective clinical trial recruitment strategies. In Greece, limited research has been conducted on this topic so far. This study aims to contribute to filling this gap.

**Methods:**

A cross-sectional study was conducted. Purposive sampling was used to select participants. The Greek version of a recently developed questionnaire measuring patient views on participation in clinical trials, a 27-item scale distributed into four factors, was tested. In addition, participants were asked to provide information regarding their socio-demographics. A demographic comparison was conducted.

**Results:**

The four-factor solution derived in our study consisted of the same 27 items and it was different from the six-factor solution that Arnetz et al. proposed. The factors risks and benefits, that consisted of 5 and 3 items respectively in the six-factor solution, were merged into one factor that consisted of 10 items in the four-factor solution. The four factors produced were Risks and benefits (ten items, α = 0,867), Patient’s expectations (six items, α = 0.864), Patient’s participation (five items, α = 0.827), and Cost and convenience (five items, α = 0,770). We found that demographic factors did not impact patients’ opinions about clinical trials participation, except for gender. The participants reported as important for participating in clinical trial: receiving clear and adequate information (95,5 %) and being given the opportunity to ask questions (97,8 %), take part in discussions regarding their own treatment (94,6 %), and voice their concerns and opinions (91,1 %). As factors strongly associated with participants’ willingness to participate in a clinical trial were reported: concerns about the risks of being in a clinical trial (87,5 %), the possible side effects of clinical trials (86,3 %), the type of treatment given in a clinical trial (83,7 %), and whether participation would improve their quality of life (QoL) (81,5 %).

**Conclusions:**

The preliminary validation of the Greek version of the questionnaire measuring patient perceptions and expectations of participating in clinical trials demonstrated acceptable validity and reliability and could be further tested in larger samples. The findings that emerged from this study are in line with previous literature.

**Supplementary Information:**

The online version contains supplementary material available at 10.1186/s12913-021-07111-x.

## Background

Recruitment of patients for participation in randomized controlled clinical trials may be challenging, causing delays in trials and hence boosting their costs [1]. It is indicative that fewer than 1 in 20 adult cancer patients enroll in cancer clinical trials. Importantly, participation to clinical trials can be impeded by numerous types of barriers, such as structural, clinical and attitudinal, while the demographic and socioeconomic background of patients also performs a crucial role [2, 3]. The workload and costs of clinical trials may be profoundly affected by these barriers. For instance, trials may close prematurely or take a longer time to complete [4].

A variety of factors have been cited in the literature as factors affecting the patient’s willingness to participate in clinical trials. For potential participants, barriers may be frequent due to ethical concerns around trials as where as the lack of clarity and complexity of providing informed consent [5]. For instance, the literature suggests that not only altruism but also self-interest may be the motivating factor for participation [6]. Furthermore, factors that have been associated with patient participation include communication with providers and family members, preference for alternative treatments, convenience, medical history, and insurance [7]. Note that, as trials are becoming increasingly complex (including translational research), many new factors have been emerged as barriers for patients to participate in clinical trials. For instance, one third of older adults in England has difficulty in reading and understanding basic health-related information, and patients can have misconceptions about research-related issues, such as the goals of the clinical trial, the risk of side effects and the likelihood of personal benefits [4, 8–10]. Moreover, mandatory research biopsies may discourage patients from participating in trials [11, 12]. To successfully conduct a clinical trial, the different attitudes and motivations of participants according to trial type must be considered [13].

However, existing research on patient views on participation in clinical trials is not yet very advanced or broad. Much of it relates to cancer patients, followed by cardiology and heart attack patients [7]. However, other medical conditions have remained unexplored. A better understanding of patients’ views of participating in clinical trials would contribute to a better execution of clinical trials. It may facilitate trial recruitment and improve patient satisfaction with the recruitment process [4]. Trial recruitment should be facilitated, provided that a significant percentage of the general population is unlikely to have a negative attitude towards participating in clinical trials. For instance, Comis et al. found that a significant percentage of Americans are willing or inclined (while holding some questions) to participate in a cancer clinical trial [14]. At any rate, incorporating patient views into the patient recruitment process can promote both patient-centered research and clinical trial effectiveness.

The aim of the present study was to translate and validate the Greek version of the questionnaire developed by Arnetz et al. (2019) for measuring patient perceptions and expectations of participating in clinical trials. We obtained permission from the authors to conduct this study.

In the absence of adequate specific empirical knowledge about this topic in Greece, we carried out a prospective patient survey using the translated questionnaire to gain a better understanding of patients’ willingness and motivations for participating in clinical trials, and their opinion on trial information and the consent process. The translated questionnaire was administered online, given current restrictions due to COVID-19, with the participation of 313 individuals. All reasonable precautions were taken to ensure the construction of a randomized sample, with limited scope for selection or self-selection bias, or any other type of bias. Notwithstanding this, due to the compensation being offered, a risk of self-selection bias could exist, with people being influenced to participate by the monetary incentive offered. Nevertheless, the amount of c. EUR 2.5 offered to participants was set after careful consideration by the research administrators. This would be seen as a fair compensation for the time spent filling the questionnaire (c.20 min are needed), while salary standards in Greece were taken into account (pro rata the amount offered would be in line minimum wage standards in Greece). Therefore, this would be unlikely to unduly influence the large majority of possible participants in Greece. Moreover, sufficient instructions and clarifications were provided to participants at the research outset from the questionnaire administrators, to ensure that this risk is minimized to the largest extent possible.

## Materials and methods

### The questionnaire

To further expand the usage of the pilot questionnaire developed by Arnetz et al. (2019) for measuring patient perceptions and expectations of participating in clinical trials, we attempted to translate, adapt, and validate that questionnaire in the Greek context, after having conducted a literature review on earlier research on patient perceptions of involvement in medical care, in general and especially in Greece.

 The translated and adapted to the Greek context questionnaire for measuring patient perceptions and expectations of participating in clinical trials was anonymously administered, with four response options per item (strongly disagree, partly disagree, strongly agree, partly agree OR very important, important, slightly important, unimportant). Demographic data was also recorded, including gender (M/F), age (years), educational background, race, marital status, professional situation, the participants’ physical and mental health status, and the participants’ medical history, including if participants had previously already participated in clinical trials.

### Translation

The questionnaire was translated according to the Medical Outcomes Trust (1997) criteria. First, three bilinguals carried out translation of the questionnaire from English into Greek. After consensus had been reached within the group of translators, two other bilinguals translated it back from Greek into English. The translated version was reviewed for item relevance and content validity by a panel of researchers experienced in quantitative research as a type of empirical investigation, at the School of Medicine (Faculty of Health Sciences, Aristotle University). Some slight changes were made to better adapt the questionnaire to the Greek context. Then, the questionnaire was reviewed by 20 senior medical students to locate any difficulties in understanding the meaning of the questions. Please note that in this paper (including the Additional Files), the items of the questionnaire have been translated from Greek (as presented in the Greek version of the questionnaire) into English.

### Participants

Patients for this study were recruited online, from 13 different districts of Greece, via links provided to patients that were visiting Greek clinic and hospital websites, in order to discuss a medical condition with a clinic or hospital representative or book an examination appointment for them or a relative. Data was collected from the visitors of websites and customer portals of large clinics and hospitals in Greece. All participants were provided with detailed information and clarifications, regarding the purpose of the study, via both email and phone conversations to ensure compliance with all ethical considerations [15]. Data collection for the questionnaire took place between January and March 2021, and 313 patients were recruited in total. All respondents willing to participate received either a EUR 2.5 compensation or a EUR 3.0 Amazon gift card for their participation. The responses were anonymized and explicit consent to participate was provided by all participants to proceed with filling in the questionnaire. Participants were assured that their personal data would be kept confidential and separate from their questionnaire responses.

### Statistical analysis

Data was analyzed using IBM SPSS Statistics for Windows, version 25.0. (Armonk, NY: IBM Corp). The agreement between responses at two time points was examined utilizing RStudio [16] and raters package [17], whereas confirmatory factor analysis was carried out utilizing lavaan [18] and semTools packages [19].

Kolmogorov-Smirnov and Shapiro-Wilks tests were conducted, as well as Q-Q plots were constructed to investigate normality in data. Means and standard deviations were calculated for interval data following the normal distribution and, median and interquartile range for non-normal or ordinal data. Test-retest reliability was evaluated by administering the questionnaire to 35 participants twice, within an interval of two weeks. Since data obtained from participants’ responses were ordinal, the non-parametric Wilcoxon test was conducted for examining the differences between the responses at each time point. Agreement between responses at the two time points was evaluated by the modified Fleiss’ Kappa, [20] since an imbalance distribution of responses was involved.

To determine the factor structure of the questionnaire, an exploratory factor analysis (EFA) was conducted using Principal Component Analysis (PCA) with Varimax rotation.

Preliminary analyses were implemented for considering the appropriateness of EFA. In particular, the Kaiser-Meyer-Olkin (KMO) measure of sampling adequacy was computed for quantifying the degree of intercorrelations among the variables and the appropriateness of factor analysis [21]. Hutcheson et al. (1999) consider values between 0.5 and 0.7 as mediocre, values between 0.7 and 0.8 as good, values between 0.8 and 0.9 as great, and values greater than 0.9 as superb [22]. The Bartlett’s test of sphericity was conducted to examine the correlation among the items [23]. To justify factor analysis, KMO values should exceed 0.60 [24] and Bartlett’s test of sphericity should be significant. The Determinant was computed for testing the presence of multicollinearity with values less than 0.0001 to be considered acceptable (no multicollinearity).

The Kaiser’s criterion [25] and scree plot [26] were utilized for determining the number of factors to extract at the initial stage of the process. Different factor structures from several trial solutions were compared, mainly by considering the proportion of total explained variance, the factor loadings and their interpretability in order to arrive at the best representation of the data.

Regarding the factor loadings, Hair et al. provide guidelines for practical significance, which indicate that a factor loading of ± 0.3 means that the item is of minimal significance, ± 0.4 indicates it is more important and ± 0.5 indicates the factor is significant [27]. Statistical significance of factor loadings was based on the sample size, with a sample size of 200–300 participants to require a factor loading from 0.29 to 0.38 [28]. Therefore, the final factor solution required a minimum factor loading of 0.4 (absolute value). The proportion of the total variance explained by the retained factors should be at least 50 % [29].

Reliability of each factor was assessed by the Cronbach’s alpha, a measure of internal consistency [30]. Values greater than 0.7 indicate a reliable factor [31].

Confirmatory factor analysis (CFA) was performed based on diagonally weighted least squares (DWLS) estimation method, which is considered more appropriate than normal theory-based maximum likelihood for ordinal data. The method was carried out to test the four-factor structure identified in the exploratory factor analysis. The goodness of fit was evaluated through the most used fit indices [32]: the chi-square test, the goodness-of-fit index (GFI), the root mean square error of approximation (RMSEA), the standardized root mean square residual (SRMR), the comparative fit index (CFI), the Tucker-Lewis index (TLI) and the normed fit index (NFI).

Convergent validity was evaluated based on the size of factor loadings the average variance extracted (AVE) and the composite reliability (CR) of each construct (factor). According to the guidelines in [27], all factor loadings should be statistically significant and the standardized loading estimates should be 0.5 or higher, and ideally 0.7 or higher. In addition, the values of AVE and CR should be at least 0.5 and 0.7 respectively. Discriminant validity was investigated through heterotrait-monotrait ratio of correlations (HTMT) with values higher than 0.85 or 0.90 to suggest issues with respect to discriminant validity.

Associations and correlations were investigated between the four factors and participants’ characteristics, such as, gender, age, education, marital status and whether participants took part in a medical research or clinical trial in the past. The values of the factors were compared between two groups of participants applying Mann-Whitney test, and correlations were investigated computing Spearman’s correlation coefficient [33]. Non-parametric tests were applied due to the results of Kolmogorov-Smirnov and Shapiro-Wilks tests, as well as the shape of Q-Q plots that suggested non-normality for the values of the factors.

Statistical significance was set at 5 % (*p *< 0.05).

## Results

The sample comprised 313 participants, 156 (49.8 %) females and 157 (50.2 %) males, aged between 18 and 76 years (M = 43.9, SD = 14.1). The participants were recruited from 13 districts of Greece and filled in the survey online.

### Test-retest reliability

Thirty-five participants completed the questionnaire in test and retest mode two weeks later. Wilcoxon tests showed no significant differences between the two tests (Supplementary Table 1) [All tables are mentioned in the Supplementary Material-Additional File 2 by their number]. The modified Fleiss’ kappa statistic (s*) indicated substantial to almost perfect agreement (0.61-1.00) for 22 out of 27 questions and moderate agreement (0.41–0.60) to 5 out of 27 questions (Supplementary Table 1). Overall, test-retest results indicated reliability of the questionnaire.

### Preliminary analysis for EFA

Exploratory factor analysis (EFA) was conducted on the 27 items in the questionnaire and 6 factors were derived based on Kaiser’s criterion (eigenvalues greater than or equal to 1). The KMO measure of sampling adequacy was 0.898 and Bartlett’s test of sphericity was significant (χ2(351) = 3917.3, *p* < 0.001), indicating that factor analysis was appropriate for this data. The Determinant was equal to 0.00002 > 0.00001 suggesting no multicollinearity. Taken together the results indicated that all 27 items in the questionnaire were suitable for inclusion in factor analysis.

### Initial factor analysis

Following the Kaiser’s criterion, EFA resulted in a six-factor solution that accounted for 62.2 % of total variance. There were two issues regarding this solution. First, there were two out of six factors with only two items loaded on each one of them. Second, examination of the scree plot (Supplementary Fig. 1) [Supplementary Material-Additional File 1] showed a sharp drop in eigenvalues from one to three factors and a slight drop from the fourth to the fifth factor. Since, Kaiser’s criterion and scree plot suggested a different number of factors, EFA was re-applied for extracting five, four or three factors. The criteria for selecting one out of these three solutions were based on factor loadings, proportion of total explained variance and interpretability of the final rotated solutions.

The five-factor solution accounted for 58.2 % of total variance, all factor loadings were above 0.4 and there were four items with cross-loadings above 0.4 on a second factor. The four-factor solution accounted for 54.0 % of total variance, one factor loading was below 0.4 and there was one item with cross-loadings above 0.4 on a second factor. The three-factor solution accounted for 49.0 % of total variance, all factor loadings were above 0.4 and there were four items with cross-loadings above 0.4 on a second factor. Regarding the interpretability, the four-factor solution identified factors that were fairly interpretable, while five-factor and three-factor solutions identified three and one interpretable factor, respectively. Based on the above findings, the four-factor solution was preferred and EFA was re-applied for extracting four factors after excluding from the sample the item with a loading less than 0.4 (item: cost-free treatment).

### Final Factor analysis

The KMO measure of sampling adequacy was 0.901 and Bartlett’s test of sphericity was significant [χ2(325) = 3810.9, *p* < 0.001], indicating adequate factorability of the items. The total explained variance was 55.3 %. The first factor, accounting for 16.2 % of the total variance, was focused on patient expectations from clinical trials. The second factor, the risks and benefits of the treatment in clinical trials accounted for 15.3 % of the total variance, while the third factor related to patients’ participation in clinical trials,and accounted for 12.3 % of the total variance. The final factor, accounting for 11.6 % of the total variance, was focused on cost and convenience of clinical trials.

The results of EFA are presented in the Supplementary Table 2 along with the assigned labels to factors. All loadings below 0.4 were suppressed. According to the results of EFA presented in the Supplementary Table 2, three factors comprised items with loadings greater than 0.50 and one factor comprised four (out of ten) items with loadings that ranged from 0.45 to 0.49.

### Internal consistency

All factors were considered reliable, with values of Cronbach’s alpha ranging from 0.770 to 0.867, and item-total correlations above 0.3 (Supplementary Table 3).

### Confirmatory Factor Analysis (CFA)

CFA was conducted on the four-factor solution derived by the EFA. To investigate the model’s goodness of fit, a number of statistics were obtained, as described in the section “Materials and Methods” above. As can be seen from Table 1, χ^2^/df (1.445), GFI (0.988) and RMSEA (0.038) values fulfilled the criteria to indicate good absolute fit. Accordingly, CFI (0.995), TLI (0.994) and NFI (0.984) values were greater than 0.950 suggesting good incremental fit. Overall, CFA results suggested that the measurement model provided a good fit.

**Table 1:** Goodness-of-Fit Statistics for the Four-Factor Model

Fit Indices	Values	Recommended Value
χ^2^	423.375	N/A
df	293	N/A
χ^2^/df	1.445	≤ 2.00
GFI	0.988	≥ 0.90
RMSEA	0.038	≤ 0.06
(90 % Confidence Interval)	(0.030,0.045)	
CFI	0.995	≥ 0.90
TLI	0.994	≥ 0.90
NFI	0.984	≥ 0.90

All of the standardized loading estimates were statistically significant (*p* < 0.001) with values higher than 0.70 with two exceptions. The items that refer to compensation and quality of life with loadings of 0.604 and 0.699 respectively. Although these values are below the ideal threshold, it was decided to be retained in order to support content validity (Table 2). The average variance extracted estimates and the composite reliabilities are shown at the bottom of Table 2. The AVE estimates ranged from 0.576 to 0.688 and CR estimates ranged from 0.870 to 0.944, both exceeding the recommended thresholds of 0.50 and 0.70 respectively. All this evidence supported the convergent validity of the measurement model.

**Table 2:** Standardized Factor Loadings, Average Variance Extracted and Composite Reliability Estimates

Items	Patient expectations	Risks and benefits	Patients’ participation	Cost and convenience
31 Understanding—help you in future	0.801			
29 Understanding—help you now	0.897			
32 Understanding—help others in future	0.813			
28 Understanding trial aim	0.848			
30 Understanding—help others now	0.870			
27 Part of medical research	0.739			
22 Risks		0.927		
21 Side effects		0.839		
20 Purpose of treatment		0.873		
19 Type of treatment		0.765		
23 Benefits		0.778		
25 Pain/discomfort		0.701		
39 Ethically approved		0.759		
38 Confidential		0.758		
26 Quality of life		0.699		
40 Person to contact during trial		0.816		
17 Involved in discussions			0.896	
16 Express views			0.803	
18 Involved in decisions			0.805	
15 Can ask questions			0.821	
14 Receive clear information			0.774	
34 Out of pocket costs				0.826
37 Need help in participation				0.740
33 Compensation				0.604
36 Travel				0.744
35 Convenient				0.856
**AVE**	0.631	0.688	0.674	0.576
**CR Reliability**	0.944	0.930	0.912	0.870

*CR*  Composite Reliability; *AVE*  Average Variance Extracted

The heterotrait-monotrait ratio of correlations (HTMT) are presented in Table 3. Correlations ranged from 0.381 to 0.784, lower than 0.85 suggesting good discriminant validity.

**Table 3:** Heterotrait-Monotrait Ratio of Correlations (HTMT)

Factors	Patient expectations	Risks and benefits	Patients’ participation	Cost and convenience
Patient expectations	-	-	-	-
Risks and benefits	0.784	-	-	-
Patients’ participation	0.381	0.403	-	-
Cost and convenience	0.716	0.708	0.382	

### Factor exploratory analysis and statistical significance

Exploratory factor analysis was conducted to determine the factor structure of the questionnaire and four factors were derived, namely, (i) patient expectations, (ii) risks and benefits, (iii) patient participation, and (iv) cost and convenience.

In the study of Arnetz et al. (2019), the pilot questionnaire consisted of 30 items and it was delivered to 53 patients. The authors derived a six-factor solution that encompassed factors concerning motivation, risks of participation, benefits of participation, understanding the purpose, cost/convenience, and contribution to health improvement. The main questionnaire of the study consisted of 27 items and it was delivered to 55 patients. The factor solution was not the same with the one derived in the pilot phase.

The final four-factor solution derived in our study consisted of 26 items and it was different from the six-factor solution that Arnetz et al. proposed. The factors risks and benefits, that consisted of 5 and 3 items respectively in the six-factor solution, were merged into one factor that consisted of 10 items in the four-factor solution. There were 7 common items in the two solutions. Also, the factor cost/convenience was almost the same in both solutions, with the four-factor solution comprising one more item than the six-factor solution. The remaining factors were significantly different between the two solutions.

Across the four factors formed (i.e. (i) patient expectations, (ii) risks and benefits, (iii) patient participation, and (iv) cost and convenience), the percentage of participants who fully or partially agreed differs statistically significantly from the corresponding percentage of those who partially or completely disagreed. This provides confidence on the validity of the results, and the recognition of the most crucial elements for each factor. Among different demographic characteristics, the most crucial, statistically significant differences were related to the participants’ gender, with statistically significant differences of views between men and women observed in most factors.

For factor (i) patient expectations (Supplementary Table 4), for each of the 6 items of the questionnaire included in this factor, the percentage of participants who fully or partially agreed differs statistically significantly from the corresponding percentage of those who partially or completely disagreed (*p* < 0.05).

Also, the statement that the patient has the ability to ask questions (the highest percentage of agreement) shows a significantly higher degree of agreement (Z = -4.888, *p* < 0.001) than the statement that the patient receives clear information (the second higher agreement rate), providing confidence on correctly identifying the most significant element of this factor.

For factor (ii) risks and benefits (Supplementary Table 5), for each of the 10 questionnaire items included in this factor, once again the percentage of participants who consider the respective issues as very or quite important differs statistically significantly from the corresponding percentage of those who consider them as little or not at all important (*p* < 0.05).

However, the issue of risks and side effects that patients may face, which was identified as the most significant element of this factor, does not differ significantly in the degree of importance declared by the participants in the study (Z = -0.784, *p* = 0.433) from the second and third most important elements. On the other hand, the risks (Z = -6.751, *p* < 0.001) and the side effects (Z = -6.518, *p* < 0.001) that patients may face differ significantly in the degree of significance declared with the degree of significance stated on whether the treatment would be provided free of charge. Therefore, this would provide us confidence in assuming that potential expenses are of comparatively lower importance for clinical trial participants.

For factor (iii) patient participation (Supplementary Table 6), for each of the 5 questionnaire items included in this factor, the percentage of participants who consider the respective expectations as very or quite significant differs statistically significantly from the corresponding percentage of those who consider them as little or not at all important (*p* < 0.05). However, no statistically significant differences were found between expectations from participation in clinical trials regarding the degree of significance reported (*p* > 0.05). Here, a potentially larger sample might be helpful in drawing more definitive results.

Finally, for factor (iv) cost and convenience (Supplementary Table 7), for each of the 5 items of the questionnaire included in this factor, the percentage of participants who consider the respective issues as very or quite important differs statistically significantly from the corresponding percentage of those who consider them as little or not at all important (*p* < 0.05).

However, the issue of confidentiality of all personal data and results of the clinical study (most important element) and the issue of possible other costs for participation in this clinical study (second most important element) do not differ significantly in the degree of importance stated by the participants in the study (Z = − 1.467, *p* = 0.142). On the other hand, the issue of confidentiality of all personal data and results of the clinical study (Z = -4,420, *p* < 0.001) and the issue of possible other costs for participation in this clinical study (Z = -3,480, *p* = 0.001) show a significant difference in the degree of significance declared for the remaining elements of this factor.

### Demographics and demographic comparison

Participants’ ages ranged from 18 to 76 years (*M* = 43.9, SD = 14.1) with 51.4 % (*n* = 161) identified as female, 48.6 % (*n* = 152) as male, 63.3 % married or partnered, approximately 30 % reported living alone (single, divorced or widower), 22.7 % reported being retired or not working (10.2 % and 12.5 % respectively), 62.6 % reported being working or self-employed 49,5 % (*n* = 155) had post-secondary and higher education, 32,9 % (*n* = 103) had secondary education, and 13,1 % (*n* = 41) possessed a Master degree. The vast majority of participants (97.8 %) identified themselves as white people. Approximately half of the participants (47.6 %) were not “high-income participants.” The mean of their net personal annual income was lower than 10,000 euros. The percentage of participants reporting net personal annual income ranging from 10,000 euros to 20,000 euros was lower (32.3 %), with 13.1 % of participants reporting net personal annual income ranging from 20,000 to 30,000 euros.

The time in minutes that participants were willing to spend for transferring to a research site for study-related procedures ranged from 0 to 400 min (*M*dn = 22.5, IQR: 10.0–30.0), while on average they were willing to cover a median distance of 10 km.

The majority (54.6 %) of participants indicated that they would choose to go to a research site by car, with 21.1 % of the participants reporting their preference to go on foot, 18.8 % by means of public transportation and 5.4 % on bicycle or motorcycle.

Slightly more than 9 out of 10 participants (91.7 %) reported having health insurance.

The vast majority of participants (80.8 %) reported their physical or psychological condition as being in a peak state (16.6 %), in a very good state (32.6 %) or just in a good state (31.6 %), with 19.2 % of participants reporting their condition as mediocre (15.7 %) or even bad (3.5 %).

Furthermore, in our study we found that 6.1 % of our respondents had previously participated in a clinical trial, with the 89.5 % of them reporting they had received a clear and direct benefit (26.3 %) or had rather been benefited (63.2 %).

The majority of our participants (61.7 %) reported they had never previously taken medications or undergone treatment for any of the diseases enquired, with a lower proportion of participants reporting they had undergone treatment for anxiety (16.0 %), hypertension (11.8 %), cardiovascular disease (10.9 %) and panic attacks (7.3 %).

The participants in our study strongly supported the view that participation in clinical trial means that the participants (patients): (a) are offered the opportunity to be clearly informed (95.6 %) and ask questions (97.8 %), (b) receive clear and adequate information (95.5 %), (c) are offered the opportunity to take part in discussions regarding their own treatment and health care (94.6 %), (d) are offered the opportunity to voice their concerns and opinions (91.1 %) and (e) are offered the opportunity to become involved in decision making processes regarding their own treatment and health care (85.6 %).

 Furthermore, our participants were asked about the importance and impact of some factors on their willingness to participate in a clinical trial aiming at curing a disease or eliminating a health-related problem. The elements that were reported as most significant by the greatest proportion of participants were: (a) the concerns about the risks of being in a clinical trial (87.5 %), (b) the possible side effects of clinical trials (86.3 %), (c) the type of treatment given in a clinical trial (83.7 %), (d) whether participation would improve their quality of life (81.5 %), with only 62.6 % of the participants finding very significant the fact that the treatment will be provided free of charge.

Almost all the participants in our study (98.7 %) had no difficulty understanding the items of the scale, with 88.2 % reporting that the administered scale was not lengthy.

Moreover, the participants in our study were asked about the elements “knowledge / information”, “their expectations towards participation in clinical trials”, “convenience” and “costs / payments.” The factors that were reported as most significant by the greater proportions of participants were: (a) the confidentiality of participants’ personal data in the clinical trial and the confidentiality of the results of the clinical trial (68.7 %), (b) the understanding of the way in which the particular clinical trial might benefit the respondent (67.7 %) and other patients at the present time (68.4 %), and (c) the understanding of the purpose of the particular clinical trial (66.8 %), with (d) 52.7 % of the participants finding very significant the eventual need for a participant to travel to a research site for study-related procedures.

Among different demographic characteristics, the most crucial, statistically significant differences were related to the participants’ gender.

Mann-Whitney tests indicated significant differences between males and females regarding views on the following factors: ii) the risks and benefits (Z = 2.574, *p* = 0.010), iii) patient participation (Z = 3.104, *p* = 0.002), and iv) cost and convenience (Z = 3.135, *p* = 0.002). Males especially appear to consider these factors less important than females.

Otherwise, none of the four factors demonstrated statistically significant correlation to age, education, or marital status (*p* > 0.05). Nevertheless, we do observe some factors emerging from our data analysis as associated with taking a certain stance on the questions included in the administered scale. More precisely, we find that the responding option “strongly agree” received greater score among participants being over 60 years old, being retired or being in married cohabitation, with the participants in very good psychological condition showing a trend towards choosing the responding option “very important.”

For factor (ii) risks and benefits, women consider as more important than men the issue of risks (Ζ = -2,198, *p* = 0.028), side effects (Ζ = -3,343, *p* = 0,001) and possible discomfort, inconvenience or pain (Ζ = -3,011, *p* = 0.003), which they may face in the context of a clinical trial.

For factor (iii) patient participation, women consider it more important than men to understand how the clinical trial could help them personally at the moment (Z = -3,430, *p* = 0.001) and in the future (Z = -2,900, *p* = 0.004), to understand the purpose of the clinical trial (Z = -2,956, *p* = 0.003) and that the clinical study will be part of a broader medical research study (Z = -1,969, *p* = 0.049).

Finally, for factor (iv) cost and convenience, women consider more important than men the need to travel to participate in the clinical trial (Z = -3,515, *p* < 0.001) and the assistance of another person to participate in the clinical trial (Z = -3,426, *p* = 0.001).

Moreover, according to Mann-Whitney results, individuals, who had participated in a medical research or clinical trial, regarded factor (ii) risks and benefits of the treatment in clinical trial (Z = 2.570, *p* = 0.010) and factor (iv) cost and convenience of clinical trials (Z = 2.936, *p* = 0.003) as less important than individuals who had not participated in a medical research or clinical trial in the past. This clearly indicates the importance of educating patients on the benefits of clinical trials, as well as providing them with clear information at the outset, to clarify their expectations.

## Discussion

We found that demographic factors did not impact patients’ opinions about clinical trials participation, with the exception of gender differences. Catania et al. found that demographic factors did not impact patients’ willingness to participate in clinical trials [34]. However, several studies reported several facilitators or significant barriers to trial participation [35, 36]. Among factors affecting people’s willingness to participate in clinical trials the following have been cited: age and race [37–40], inconvenience to participate [41, 42], economic benefit [43, 44], socio-economic factors [37, 45], education level [37, 44, 46], availability of treatment options, participants’ burden, participants’ health status, racial difference [47], and types of research [48].

Participants’ gender has been cited in literature as a factor affecting people’s willingness to participate in clinical trials [37, 41, 49, 50]. Several studies have reported that women were less likely than men to enroll in clinical trials [40, 51–54]. However, some studies found higher clinical trial participation rates among women [55, 56], with a study reporting “no evidence of any systematic under-representation of women in clinical trials” [57]. A population-based study found no evident gender differences in clinical trial participation [41].

Sufficient and clear information, discussion with researchers, possible side effects in clinical trials and potential QoL-related benefits of participating in clinical trials have been reported as factors that are very important for participating in clinical trials.

### Information

Sufficient and clear information is a strong motivation to participating in a clinical trial whereas lack of information or explanation is a strong motivation to refuse participation in a clinical trial [58, 59]. While it is paramount to ensure that patients can make an adequately informed decision about participation in clinical trials [60], the consent to participate on a clinical trial often is not adequately informed [37]. In a study conducted by Mowlabaccus and Jodheea-Jutton, 19.1 % of the respondents were of the view that participants in clinical trials often are not adequately informed [37]. In a study conducted by Godskesen et al. it has been cited that a minority (20–28 %) of patients felt that the amount of information provided was adequate [9]. Limited previous information about clinical trials is cited as an important barrier to participation [61]. Note, however, that providing adequate information to clinical trial participants is not an easy task. As is the case with patient clinical information, clinical trial information should be concise. Lengthy information can be confusing for patients [62]. Furthermore, trial information should be tailored according to the individual patient’s core values and preferences [63]. Importantly, it has been cited in literature that patients may have misunderstood the purpose of a clinical trial [4, 64] or the potential personal benefit from a clinical trial [65]. Patients who enter quickly into clinical trial registers may not feel that they fully understood the implications of their clinical trial participation [66]. Therefore, patients should be given an adequate time frame to consider the information provided [4]. At any rate, adequate information is a strong factor promoting clinical trial participation. A majority (76 %) of the respondents in a survey conducted by Catania et al. “considered that a thorough explanation of toxicity/safety of the proposed treatment would lead them to a more conscious motivation in deciding” [34].

Several studies suggest that patients who participated in clinical trials should be offered the trial results [4, 62, 67–69]. Not receiving results may discourage patients from participating in future trials [67]. The majority of participants in a survey conducted by Jones et al. valued the publication of trial results as either important (36 %) or very important (48 %). Moreover, a majority (63 %) reported that the public release of prior study results would be a crucial motivating factor to participate in trials. Importantly, 85 % argued that the receipt of information on the publication track record of research sponsors would be seen as important or very important [60]. In this perspective, Jones et al. state that the public release of trial results may be a factor motivating clinical trial participation [60]. Interestingly, Cox et al. state that certain patients are purely focused on their personal results in the study, others look mostly at the overall study results, but many are actually interested in both of these [69].

Moorcraft et al. arguably state that many patients consider the receipt of the trial results as a sign of respect for their participation and contribution to the trial [4]. The potential distress of hearing bad news is not a sufficient reason for not informing trial participants of the trial results [69].

### Discussion with researchers

Participating in discussions and engaging in shared decision-making processes may improve the participant–research team relationship by creating a research environment in which the patients feel that they are valued and respected.

Participants’ trust in researchers has been cited in literature as a factor affecting their willingness to participate in clinical trials [4, 9, 14, 62, 65, 70–75]. Trust in researchers is a strong facilitator of clinical trial participation. Catania et al. found that trust in researchers (76 %) or in the institute (64 %) might be facilitators of participation in clinical trials [34]. Establishing trust in health providers and creating a relationship between participants and recruiters can promote participation in clinical trials [76]. Catania et al. reported that 31 % of their respondents were concerned by potential conflicts of interest between participants and researchers. 28 % of respondents feared that researchers were only looking to promote their own research goals [34].

Respondents (28.4 %) in a study carried out recently by Mowlabaccus and Jodheea-Jutton raise concerns towards trust in pharmaceutical companies [44]. It seems to be important to participants in clinical trials to be sure that the final beneficiaries of trials are patients rather than commercial pharmaceutical companies [77]. Furthermore, 32.8 % of the responders perceived government as an insufficient guarantor of public against unethical clinical research [44]. In the same vein was a study carried out by Burt et al. [78]. Enhancing transparency in the conduction of clinical trials can increase the trust of participants [79]. The same holds true for the procedure of obtaining informed consent [74]. Moreover, researchers’ adherence to research ethics, i.e. maintaining good ethical standards and hence reducing risk, maximizing research benefits, and assuring distributive justice to participants, contributes to promoting participants’ trust to researchers [36]. Conversely, several studies showed that financial conflicts of interest between participants and researchers can undermine participants’ trust in researchers [75, 80–84].

### Benefits

Improving participants’ health status is cited as a facilitator of clinical trial participation [44]. Ferrell et al. found that patients inclined to participate “had no other treatment options if they wanted to live longer, or they wanted to help future patients with cancer. Most believed that participation would improve or stabilize their illness and quality of life. They believed that, when the clinical trial ended, there would be new treatments” [85]. Catania et al. found that hope for having one more chance to cure (78 %) might be a facilitator of participation in clinical trials [34]. Moorcraft et al. found that 52 % of patients were motivated by a belief that they would receive the best treatment option available, closer monitoring and better quality care [4]. Pride in participating and hope for a better quality of life are motivations to consent to participating in a clinical trial [59]. The aforementioned findings are in line with other studies [9, 10, 14, 62, 65, 71, 72, 86].

### Fears

Fears of mistreatment, potential side effects from little-known drugs and exploitation have been reported in literature as strong barriers to clinical trial participation [37, 59, 87–91]. More particularly, fears of drug-related side effects have been cited in literature as a major barrier to participating in clinical trials [37, 45, 92]. In a recent study, around 47 % of participants were afraid of potential side effects [44]. Catania et al. found that 50 % of the respondents in their study were feared of receiving unknown or little-known drugs [34]. Being treated as ‘guinea pigs’ (36 %) are reported by the authors as a barrier to participation [34]. In a recent study, 35 % of the respondents were feared of being treated as ‘guinea pigs’ [44]. Several participants in a single-center study conducted in the US by Sood et al. felt that their safety (93 %) would be ensured to participate in a clinical trial [67].

### Remuneration

The vast majority of the participants in our study (89,2 %) indicated that possible remuneration for participation in clinical trial was viewed as a very or fairly significant factor. Mowlabaccus and Jodheea-Jutton found that 41 % of respondents stated that they would be motivated by a payment offer to participate in clinical trials [44]. Walsh and Sheridan found that payment is a strong facilitator of participating in clinical trials [43]. However, Manton et al. reported that participation in clinical trials is motivated not only by financial gains but also by many other motivations, included altruism [42]. Stocks et al. reported that money did not influence participation in clinical trials [93]. Furthermore, in the context of minority participation, remuneration was found to be important to male participants whereas the researcher-participant relationship was important to female participants [94].

## Limitations

As the data of our survey were collected from 13 different districts of Greece, the demographics of participants may be representative of the general population, at least to a large extent. This can be regarded as a chief strength of the study. In this survey, the respondents (patients) came from a broad range of demographics, such as educational background, age, gender, marital status, employment status. The diversity in our survey may be regarded as one of its strengths. However, while the vast majority of previous studies on the topic of patient views on clinical trial participation have used methods involving disease-specific patient groups (groups of patients with the same or similar diagnosis or symptoms), in our study any given medical condition had an equal probability of being involved. As a consequence, the results from this cross-sectional study were subject to limited disease-related bias compared to other studies involving patients with a particular medical condition. However, as the patients’ views on clinical trial participation may be strongly affected by their actual medical condition, larger and diverse nation-wide samples would enable more robust validity testing of the questionnaire and produce more generalizable results on a national level. At any rate, there is the potential for response bias. Our respondents might be patients with a particular interest in medical research. Furthermore, while the questionnaire covers a broad range of topics, it does not include questions asking “how important it would be to you what is the expected success rate of the trial” or questions exploring respondents’ willingness to participate in a clinical trial, their motives or whether they wish to know their personal (or the overall study) results of trials in which they participated. Moreover, patients’ responses may be influenced by ‘social desirability.’ It is uncertain whether patients’ responses truly reflect their views. These are limitations of our study. Ultimately, consistent with the nature of surveys is the following limitation: Quantitative research cannot capture all dimensions of the phenomenon of interest. When combined with qualitative research studies, qualitative studies may provide much more insightful understanding of patients’ attitudes towards participating in clinical trials. Despite these study limitations, the Greek version of the questionnaire can be useful for measuring the patient views of participation in clinical trials in Greece, where quantifying of these views is in its very infancy.

## Conclusions

The preliminary validation of the Greek version of the questionnaire measuring patient perceptions and expectations of participating in clinical trials demonstrated acceptable validity and reliability and could be further tested in larger samples. The findings emerged from this study are in line with previous literature. The four-factor solution derived in our study consisted of the same 27 items and it was different from the six-factor solution that Arnetz et al. proposed. The factors risks and benefits, that consisted of 5 and 3 items respectively in the six-factor solution, were merged into one factor that consisted of 10 items in the four-factor solution, Furthermore, we found that demographic factors did not impact on patients’ opinions about clinical trials participating, with the exception of gender differences. Sufficient and clear information, discussion with researchers, possible side effects in clinical trials and potential QoL-related benefits of participating in clinical trial have been reported as factors that are very important for participating in clinical trials.

## Supplementary information

**Additional file 1**

**Additional file 2**

## Data Availability

The datasets used and analysed in this study are available from the corresponding author on reasonable request.

## Abbreviations

EFA: Exploratory factor analysis
KMO: Kaiser-Meyer-Olkin
PCA: Principal Component Analysis
