# Supplementary material for: Translation and validation of the greek version of a questionnaire measuring patient views on participation in clinical trials
Source: BMC Health Serv Res. 2021 Oct 22;21:1135. doi: 10.1186/s12913-021-07111-x (PMC8530543; doi:10.1186/s12913-021-07111-x)
Supplement: Supplementary file 1 — Additional file 1 [file 12913_2021_7111_MOESM1_ESM.docx]

**Supplementary Material**

**Additional File 1 (Diagram)**


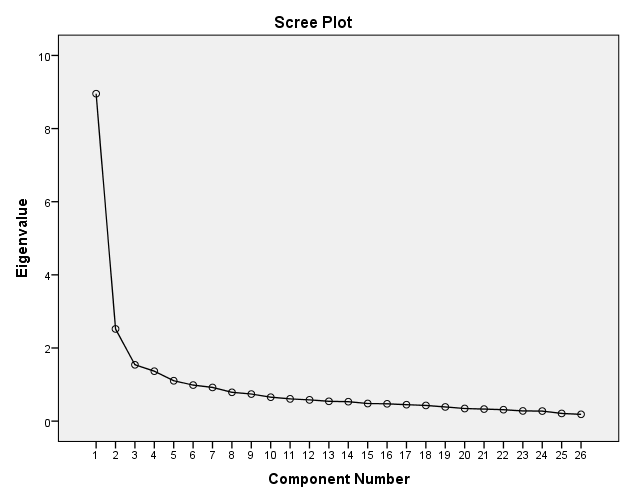


**Fig. 1: Scree plot of principal component analysis of questionnaire between eigenvalue and principal components (factors)**
