# Supplementary material for: Translation and validation of the greek version of a questionnaire measuring patient views on participation in clinical trials
Source: BMC Health Serv Res. 2021 Oct 22;21:1135. doi: 10.1186/s12913-021-07111-x (PMC8530543; doi:10.1186/s12913-021-07111-x)
Supplement: Supplementary file 2 — Additional file 2 [file 12913_2021_7111_MOESM2_ESM.docx]

**Supplementary Material**

**Additional File 2 (Tables)**

**Table 1. Test-Retest results: Wilcoxon tests and modified Fleiss' kappa statistic (s*) with percentile bootstrap confidence intervals**

| **Abbreviated item label** | **Z** | **p-values** | **s*** | **LCL** | **UCL** |
| --- | --- | --- | --- | --- | --- |
| Receive clear information | -1.311 | .190 | .749 | .588 | .886 |
| Can ask questions | -1.232 | .218 | .703 | .543 | .840 |
| Express views | -0.215 | .830 | .611 | 451 | .749 |
| Involved in discussions | -0.447 | .655 | .589 | 451 | .726 |
| Involved in decisions | -0.693 | .488 | .497 | 314 | .680 |
| Type of treatment | -0.816 | .414 | .863 | 749 | .954 |
| Purpose of treatment | -0.905 | .366 | .794 | .657 | .909 |
| Side effects | 0.000 | 1.000 | .817 | .680 | .931 |
| Risks | -0.513 | .608 | .771 | 611 | .909 |
| Benefits | -1.232 | .218 | .703 | .520 | .840 |
| Cost-free treatment | -0.500 | .617 | .680 | .520 | .817 |
| Pain/discomfort | -0.816 | .414 | 863 | .749 | .954 |
| Quality of life | -0.632 | .527 | .817 | .680 | .931 |
| Part of medical research | -1.265 | .206 | .771 | .657 | .886 |
| Understanding trial aim | -0.243 | .808 | .657 | .520 | .794 |
| Understanding—help you now | -1.155 | .248 | .726 | .611 | .840 |
| Understanding—help others now | -1.147 | .251 | .611 | .451 | .749 |
| Understanding—help you in future | -1.376 | .169 | .520 | .337 | .703 |
| Understanding—help others in future | -0.471 | .637 | 634 | .497 | .771 |
| Compensation | -0.786 | .432 | .543 | .360 | .703 |
| Out of pocket costs | -0.333 | .739 | .794 | .680 | .909 |
| Convenient | -1.387 | .166 | .749 | .611 | .863 |
| Travel | -0.881 | .378 | .634 | .451 | .771 |
| Need help in participation | 0.000 | 1.000 | .589 | .429 | .749 |
| Confidential | -1.705 | .088 | .680 | .497 | .840 |
| Ethically approved | 0.000 | 1.000 | .771 | .611 | .909 |
| Person to contact during trial | -0.420 | .675 | .657 | .451 | .817 |

**Table 2. Loadings of items on factors emerging from rotated component matrix**

| **Abbreviated item label** | **Factors** | | | | **Communalities** |
| --- | --- | --- | --- | --- | --- |
|  | **Risks and benefits** | **Patient’s expectations** | **Patient’s participation** | **Cost and convenience** |  |
| Risks | .842 |  |  |  | .752 |
| Side effects | .809 |  |  |  | .696 |
| Purpose of treatment | .609 | .400 |  |  | .574 |
| Type of treatment | .590 |  |  |  | .517 |
| Benefits | .557 |  |  |  | .481 |
| Pain/discomfort | .554 |  |  |  | .429 |
| Ethically approved | .490 |  |  |  | .425 |
| Confidential | .475 |  |  |  | .456 |
| P of life | .465 |  |  |  | .340 |
| Person to contact during trial | .453 |  |  |  | .461 |
| Understanding—help you in future |  | .750 |  |  | .630 |
| Understanding—help you now |  | .717 |  |  | .665 |
| Understanding—help others in future |  | .706 |  |  | .583 |
| Understanding trial aim |  | .699 |  |  | .622 |
| Understanding—help others now |  | .693 |  |  | .625 |
| Part of medical research |  | .553 |  |  | .434 |
| Involved in discussions |  |  | .812 |  | .699 |
| Express views |  |  | .796 |  | .657 |
| Involved in decisions |  |  | .765 |  | .629 |
| Can ask questions |  |  | .716 |  | .555 |
| Receive clear information |  |  | .674 |  | .512 |
| Out of pocket costs |  |  |  | .726 | .622 |
| Need help in participation |  |  |  | .678 | .532 |
| Compensation |  |  |  | .639 | .433 |
| Travel |  |  |  | .621 | .497 |
| Convenience |  |  |  | .581 | .556 |

**Table 3. Patient questionnaire subscales (factors) with reliability estimates**

| **Subscales** | **Number of items** | **Cronbach’s alpha** | **Corrected Item-Total Correlation** |
| --- | --- | --- | --- |
| Risks and benefits | 10 | .867 | .468-.720 |
| Patient’s expectations | 6 | .864 | .548-.732 |
| Patient’s participation | 5 | .827 | .535-.725 |
| Cost and convenience | 5 | .770 | .404-.633 |

**Table 4.** Participant information and involvement in the procedure of a clinical trial

|  | **Strongly agree** | **Slightly agree** | **Slightly disagree** | **Strongly disagree** |
| --- | --- | --- | --- | --- |
| The participant receives clear information | 65.8 | 29.7 | 3.8 | 0.6 |
| The participant is offered the opportunity to ask questions | 79.9 | 17.9 | 1.6 | 0.6 |
| The participant is offered the opportunity to voice his or her concerns and views | 63.9 | 27.2 | 6.7 | 2.2 |
| The participant is offered the opportunity to take part in discussions about his or her treatment and care | 65.2 | 29.4 | 4.5 | 1.0 |
| The participant is offered the opportunity to get involved in decision making processes regarding their own treatment and care | 53.0 | 32.6 | 10.9 | 3.5 |

**Table 5.** Patients’ views on the treatment provided in a clinical trial (%)

|  | **Very significant** | **Fairly significant** | **Slightly significant** | **Insignificant** |
| --- | --- | --- | --- | --- |
| The risks of taking part in a clinical trial | 87.5 | 10.5 | 1.9 | 0.0 |
| The possible side effects of a clinical trial | 86.3 | 12.1 | 1.3 | 0.3 |
| The type of treatment provided | 83.7 | 14.4 | 1.9 | 0.0 |
| Participants’ quality of life might be improved | 81.5 | 16.3 | 1.6 | 0.6 |
| The aim of the treatment provided | 78.6 | 20.1 | 1.3 | 0.0 |
| Possible discomfort, inconvenience or pain related to the participation in a clinical trial | 78.0 | 17.3 | 4.2 | 0.6 |
| Possible benefits that participants may receive from their participation | 77.0 | 19.2 | 2.6 | 1.3 |
| The treatment will be provided free of charge | 62.6 | 31.0 | 4.8 | 1.6 |

**Table 6.** Participants’ Information provided and expectations related to participation in clinical trials (%)

|  | **Very significant** | **Fairly significant** | **Slightly**  **significant** | **Insignificant** |
| --- | --- | --- | --- | --- |
| The clinical trial will be part of a broader medical research study | 62.3 | 34.8 | 1.9 | 1.0 |
| Understanding the purpose of the clinical trial | 66.8 | 30.0 | 2.9 | 0.3 |
| Understanding how the clinical trial might help you at the moment | 67.7 | 27.8 | 4.5 | 0.0 |
| Understanding how the clinical trial might help other patients at the moment | 68.4 | 28.8 | 2.6 | 0.3 |
| Understanding how the clinical trial might help you in the future | 66.1 | 30.4 | 3.5 | 0.0 |
| Understanding how the clinical trial might help other patients in the future | 67.1 | 30.4 | 2.2 | 0.3 |

**Table 7.** Participants’ Information provided and expectations related to convenience and cost / remuneration (%)

|  | **Very significant** | **Fairly significant** | **Slightly significant** | **Insignificant** |
| --- | --- | --- | --- | --- |
| Possible remuneration for your participation in the clinical trial | 53.4 | 35.8 | 9.6 | 1.3 |
| Eventual additional expenses related to your participation in the clinical trial | 61.0 | 33.9 | 4.8 | 0.3 |
| Convenience of your transportation to a research site | 55.0 | 38.7 | 5.1 | 1.3 |
| Eventual need for you to travel because of your participation in a clinical trial | 52.7 | 34.8 | 10.2 | 2.2 |
| Assistance received by another person in order to facilitate your participation in a clinical trial | 55.3 | 31.9 | 11.2 | 1.6 |
| Confidentiality of your personal data and results of the study | 68.7 | 25.2 | 5.1 | 1.0 |

**Table 8.** Participants’ Information provided and expectations related to ethics and convenience of participation in the survey (%)

|  | **Very significant** | **Fairly significant** | **Slightly significant** | **Insignificant** |
| --- | --- | --- | --- | --- |
| The clinical trial is ethically approved and is under the control of the National Drug Organization | 79.6 | 17.6 | 2.6 | 0.3 |
| The possibility to ask questions to a person tasked with this survey while participating in it | 74.4 | 22.4 | 2.9 | 0.3 |
